# Neonatal screening for isovaleric aciduria: Reducing the increasingly high false‐positive rate in Germany

**DOI:** 10.1002/jmd2.12345

**Published:** 2022-10-28

**Authors:** Simona Murko, Asra Dadkhah Aseman, Friederike Reinhardt, Gwendolyn Gramer, Jürgen Günther Okun, Ulrike Mütze, René Santer

**Affiliations:** ^1^ Newborn Screening and Metabolic Laboratory, Department of Pediatrics University Medical Center Eppendorf Hamburg Germany; ^2^ Division of Child Neurology and Metabolic Medicine, Dietmar Hopp Metabolic Center, Center for Child and Adolescent Medicine Heidelberg University Hospital Heidelberg Germany

**Keywords:** isovaleric aciduria, IVA, newborn screening, pivaloylcarnitine, second‐tier, UPLC‐MS/MS

## Abstract

Newborn screening (NBS) for isovaleric acidemia (IVA) is performed by flow injection tandem mass spectrometry quantifying C5 carnitines (C5). Isovalerylcarnitine, however, is isomeric with pivaloylcarnitine which can be present in blood due to maternal use of pivaloylester‐containing antibiotics, available in Germany since late 2016. During a 36‐month period (January 19–December 21), all newborns screened in Hamburg with a C5 above cutoff (NeoGram®: 0.50 μmol/L or Neobase®2: 0.45 μmol/L) were included in the study. As a second‐tier test, a simple ultra performance liquid chromatography‐tandem mass spectrometry (UPLC‐MS/MS) method was developed to differentiate the C5 isomers pivaloyl‐, 2‐methylbutyryl‐, isovaleryl‐, and valerylcarnitine. Out of 156 772 newborns tested, one turned out to have genetically proven IVA while 99 were false positive (C5: 0.5–8.2 μmol/L) due to the presence of pivaloylcarnitine. These cases have increased year by year and show local clusters. Retrospective analysis of another 39 cases from 287 206 neonates tested at the NBS center in Heidelberg with C5 elevation (0.9–10.6 μmol/L) but clinical and biochemical exclusion of IVA yielded evidence of pivaloylcarnitine in all cases. Inclusion of a second‐tier test into NBS significantly reduces the high and increasing false‐positive rate of IVA screening. This avoids further diagnostic steps, prevents unnecessary stress and anxiety of parents in a remarkably high number of cases. If Hamburg data of 2021 are extrapolated to all of Germany, one can assume around 800 (1‰) false‐positive cases in comparison to an average of two classic IVA cases per year. Unless licensing of pivaloylester‐containing drugs for use during pregnancy is reconsidered, a second‐tier test for C5 determination is indispensable.

**Synopsis**

We suggest a simple second‐tier method to reduce the high and increasing false‐positive rate of isovaleric acidemia newborn screening in Germany due to licensing of a pivaloyl‐containing antibiotic during pregnancy.

## 1 INTRODUCTION

Isovaleric acidemia (IVA, OMIM no. 243500), a defect in leucine metabolism first described in 1966,^1^ is caused by a deficiency of isovaleryl‐CoA dehydrogenase (IVD), resulting in accumulation of isovalerylcarnitine, isovalerylglycine, isovaleric acid, and 3‐hydroxyisovaleric acid. Very early on, a distinction was made between severe (classic) and attenuated (mild) IVA which became significant with the introduction of newborn screening (NBS), which also detects mild cases that never become clinically symptomatic. Mild IVA in most cases is caused by a common missense variant of the *IVD* gene (NM_002225.5: c.932C>T; p.Ala311Val, also known as Ala282Val when referring to the mature protein). Classic IVA, if untreated, can cause coma and death due to neonatal hyperammonemic encephalopathy and severe metabolic acidosis. Therefore and because IVA is a treatable condition, early detection by neonatal screening is of utmost importance.

In Germany, IVA was part of pilot NBS studies since 1998 and has been included in the national NBS disease panel since 2005.^2^, ^3^ Prevalence of IVA in Germany as calculated from the national screening reports of 2010–2019 is 1:87 000^4^ with about 15% of cases with the severe form.^3^

NBS for IVA is performed by flow injection tandem mass spectrometry (MS/MS, without a chromatographic column) quantifying C5 carnitines. Isovalerylcarnitine, however, is only one of several acylcarnitines with a five carbon chain‐length and isomeric with pivaloylcarnitine which can be present in blood due to maternal use of pivalic acid ester‐containing medications. A false‐positive screening result due to that mechanism was first reported as early as 1998 in a single newborn from a mother treated with a pivoxilsulbactam‐containing antibiotic.^5^ False‐positive IVA screening in German newborns was first reported to the Drug Commission of the German Medical Association in 2019,^6^ 3 years after approval of pivmecillinam, a pivalic acid‐bound β‐lactam antibiotic prodrug, on the German market.

Because of a recent massive increase of false‐positive NBS results for IVA in Germany, we report here a systematic study on its occurrence in two German screening centers during a study period of 36 months and we present our UPLC‐MS/MS methods for differentiation of C5 carnitines to circumvent this problem.

## 2 MATERIALS AND METHODS

LC/MS grade water (J.T. Baker®, VWR International, Darmstadt), acetonitrile (ChemSolute®, Th. Geyer GmbH & Co. KG, Renningen, Germany), and formic acid (SigmaAldrich®, Merck, Darmstadt, Germany) were used for analysis. NeoGram® derivatized MS/MS kit and NeoBase®2 nonderivatized MS/MS kit for detection of amino acids and acylcarnitines by tandem mass spectrometry were purchased from PerkinElmer (Rodgau, Germany). The acylcarnitines pivaloylcarnitine, 2‐methylbutyrylcarnitine, isovalerylcarnitine, and valerylcarnitine were purchased from the Organic Acid Synthesis Laboratory, Dept. of Clinical Chemistry, University Medical Centers Amsterdam, The Netherlands. Internal standard (d9‐isovalerylcarnitine) was included in the MS/MS kits.

A stock solution of all four isomers (mixture of standards) with a concentration of 1 mg/ml for each component was prepared in water, further diluted, and EDTA blood was spiked to final concentrations of 0 (blank), 1, 2, 5, 10, 20, and 40 μmol/L. Then, 40 μl of blood was spotted out onto filter paper (903 Five Spot Blood Cards, Whatman®, PerkinElmer, Rodgau, Germany) and dried at room temperature. As a control and retention time marker a mixture of standards with a concentration of 2.0 μmol/L was used. The hardware configuration included a Xevo triple quadrupole mass spectrometer equipped with ESI source (Waters, Eschborn, Germany) operating in positive mode at a capillary voltage of 3.5 kV. Desolvation temperature was 500°C and gas flow 1000 L/h. Optimized cone voltage was 28 V and collision energy 20 V. Dwell time was 50 ms.

Chromatography was performed on a Waters Acquity UPLC. An UPLC BEH Waters C18 1.7 μm, 2.1 × 50 mm column was used for separation of isomers at 40°C. The chromatography was performed using solvent A (water with 0.1% formic acid) and solvent B (acetonitrile with 0.1% formic acid). NeoGram® derivatized kit and NeoBase®2 nonderivatized kit gradient conditions are presented in Tables S1 and S2, respectively.

Both methods were used for the regular neonatal screening of the 156 772 newborns tested in Hamburg during the 36‐month study period (January 2019 to December 2021), the derivatized kit during the first 15 months and the method without derivatization thereafter. If C5 was above cutoff (NeoGram®: 0.50 μmol/L; Neobase®2: 0.45 μmol/L), the same samples already prepared and analyzed by the flow‐injection method were used for differentiation of isomers. For this second‐tier method samples were simply diluted 1:10 with acetonitrile (5%). The blood spot standard (mixture of all four isomers) was injected regularly to ensure reliability of chromatography and to confirm the retention time of each isomeric compound. For validation of the method, samples of patients with genetically confirmed IVA (*n* = 9) and 2‐methylbutyrylglycinuria (*n* = 1) were analyzed.

Around the end of the study, retrospective analysis of another 39 cases detected during the study period among 287 206 screened newborns of a study population (NBS pilot study NGS2020/2025) of the screening laboratory at Heidelberg University Hospital was performed to evaluate the significance of the problem in other regions of Germany. These were newborns with C5 elevation (0.9–10.6 μmol/L, cutoff 0.63 μmol/L) in whom IVA was excluded clinically and biochemically. Determination of C5 was performed using the MassChrom® kit for analysis of amino acids and acylcarnitines, Nr. 57 000, nonderivatized (Chromsystems Instrument and Chemicals GmbH, Graefelfing, Germany).

## 3 RESULTS

### 3.1 Second‐tier test for C5 differentiation

**FIGURE 1:**
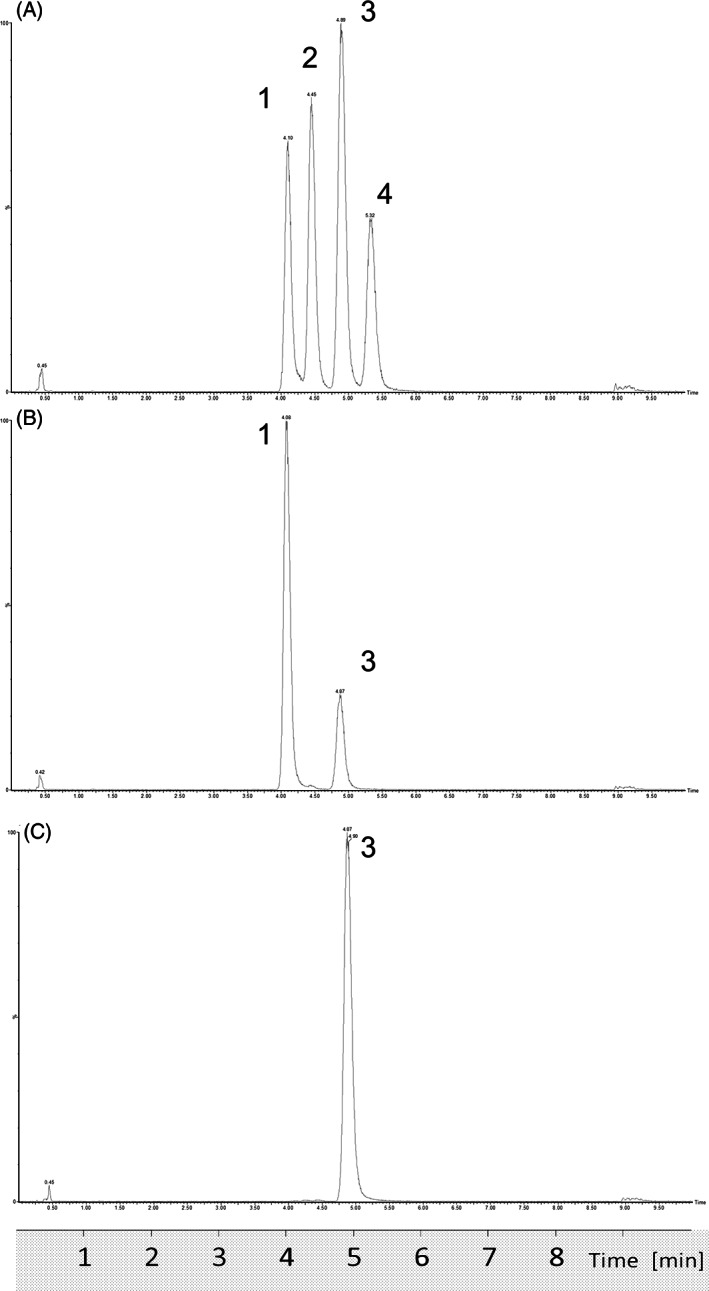
Chromatograms of (A) mixture of standards, (B) false‐positive case, and (C) genetically confirmed isovaleric acidemia patient. Peak numbers refer to pivaloylcarnitine (1), 2‐methylbutyrylcarnitine (2), isovalerylcarnitine (3), and valerylcarnitine (4), respectively.

Excellent separation of pivaloyl‐, 2‐methylbutyryl‐, isovaleryl‐, and valerylcarnitine was obtained by our second‐tier methods (Figure 1), regardless of application of the method to derivatized or nonderivatized samples (data not shown). Recovery for all C5 isomers was high, the assay showed good linearity (*R* ^2^ > 0.998 for all compounds) and low intra‐assay and interassay variability. Details of validation data are presented in Table S3.

### 3.2 Detection of pivaloylcarnitine

The number of cases with pivaloylcarnitine detection but an unremarkable isovalerylcarnitine peak in the chromatogram, that is, false‐positive cases of IVA screening found in Hamburg, increased dramatically from 20 cases in 2019 to 53 cases in 2021. A total of 100 newborns out of 156 772 tested during the 3‐year study period showed an elevated C5 concentration, of whom one was a genetically proven classic IVA case (C5 7.7 μmol/L; *IVD* c.1240C>T homozygous) while in the other 99 pivaloylcarnitine was detected as the only cause for C5 elevation. Contact with mothers was possible in more than 80% of cases and always confirmed intake of antibiotics at the end of pregnancy (although the exact trade names and the indication for antibiotic administration were often no longer known to the mothers). C5 concentrations ranged from 0.5 to 8.2 μmol/L, more than half of the cases (56.6%) showed a C5 concentration above 1.0 μmol/L. A remarkable proportion of 12.1% of cases showed a C5 concentration in the range observed in classic IVA (C5 > 4.3 μmol/L).^3^ Birth weight of false‐positive cases was in the range from 500 to 4800 g; among them 26% were premature newborns (<37 weeks of gestation) and 26% had a birth weight < 2500 g.

**FIGURE 2:**
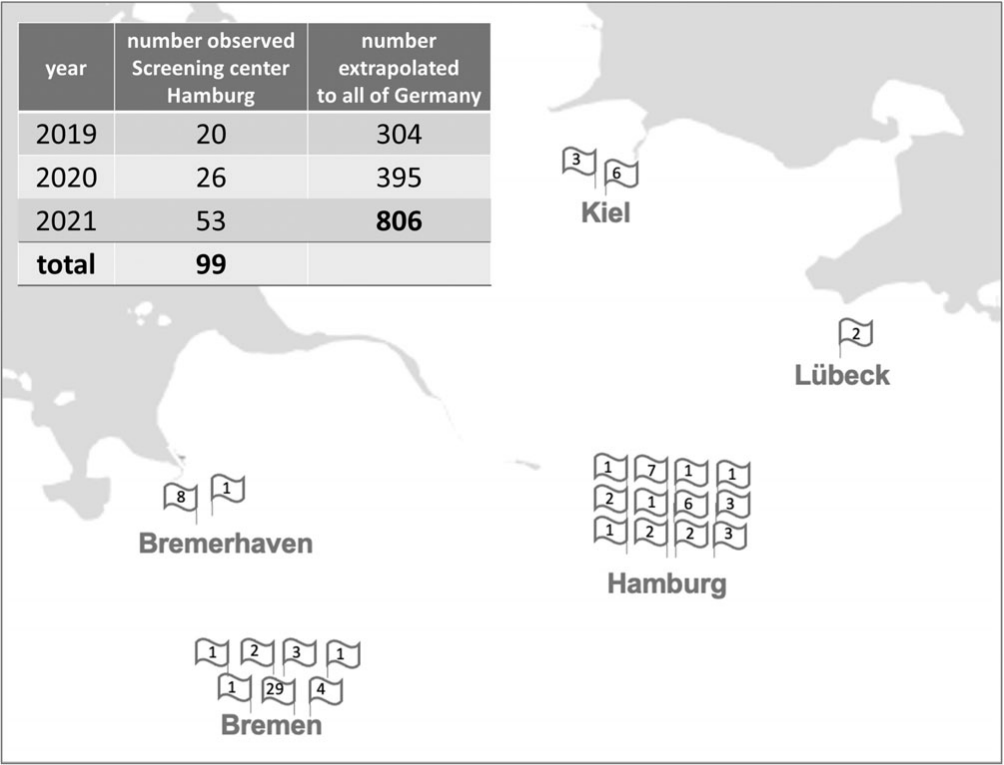
False‐positive cases of isovaleric acidemia in Northern Germany. The inserted table shows the number of cases detected over time in Hamburg and extrapolated numbers for all of Germany (birth number in 2021: 795500). The map shows the regional distribution of these cases in big cities and different obstetric units in Northern Germany (single cases from small cities were not considered for this presentation).

False‐positive cases showed local clusters (Figure 2) with 41 out of 99 (41.4%) originating from the city of Bremen, where only 13.7% of the investigated newborns were born. From these cases, 29 of 41 (72.5%) originated from one single maternity hospital.

Notably, 7.1% of all cases showed a free carnitine concentration (C0) below reference range (i.e., for NeoGram® below 10 μmol/L, for Neobase®2 below 6.3 μmol/L).

In order to interpret our results of false‐positive IVA cases, we compared our data with results from samples from the NBS laboratory in Heidelberg. Retrospective analysis of 39 cases with C5 elevation which had not resulted in a diagnosis of IVA on the basis of clinical and biochemical investigations yielded detection of pivaloylcarnitine by our second‐tier method in all cases. C5 concentrations here were in the range from 0.9 to 10.6 μmol/L. Like in Hamburg, the number of false‐positive cases detected in Heidelberg markedly increased from four cases in 2019 to 21 cases in 2021 without a single confirmed IVA in this screening cohort.

## 4 DISCUSSION

The compound of interest for the identification of IVA cases is isovalerylcarnitine as the relevant pathognomonic marker. The flow injection method used for NBS, however, is generally not able to separate interfering isomeric and isobaric compounds. Therefore, the results reported from standard NBS are the sum of all C5 isomers and isobars. It is known that exogenous sources of pivalic acid and the subsequently formed isomeric compound pivaloylcarnitine can cause a false‐positive result,^7^ and therefore, methods to differentiate pivaloyl‐ from isovalerylcarnitine in NBS both for derivatized^8^, ^9^ and nonderivatized^10^, ^11^ samples have been suggested in the past. Pivalic acid containing antibiotics have caused trouble in NBS before and problems in other parts of Europe have been reported.^11^, ^12^ Similar to antibiotics and as well for pharmacokinetic reasons, leukocyte elastase inhibitors have been bound to pivalic acid and have caused false‐positive IVA screening.^13^ Pivalic acid derivatives are also used by the cosmetic industry as emollient under the term “neopentanoate” and neopentanoate‐esters in a nipple‐fissure unguent that was routinely provided to breastfeeding mothers resulted in a high number of false‐positive screening cases in Belgium.^8^

Until very recently, when pivaloyl‐containing antibiotics have been licensed in Germany and recommended in national guidelines on treatment of uncomplicated urinary tract infections during pregnancy,^14^ this phenomenon has not been observed in Germany. Soon thereafter, however, a first report appeared^6^ which prompted us to systematically investigate the consequences for the national screening program. Remarkable numbers of false‐positive C5 elevations were observed with a rapidly increasing frequency in our screening center. The frequency of false‐positive results due to pivaloylcarnitine seems to be directly influenced by the prescription pattern of obstetricians since it varies geographically as demonstrated by the very inconsistent results in different obstetric departments that send samples to the Hamburg lab. Likewise, at the moment Northern Germany has a higher false‐positive rate than the Heidelberg lab (South‐West of the country) but in both screening centers a trend to higher numbers is observed year by year. If Hamburg data of 2021 are extrapolated to all of Germany with a birth rate of 795 500 in that year,^15^ one can calculate a number of about 800 (1‰) false‐positive cases in comparison to an average of 2 classic IVA cases per year.^3^, ^4^

Although reducing this high false‐positive result of IVA screening was the primary goal of our efforts, a second noteworthy aspect persists even with the introduction of a second‐tier test. A significant number of the newborns in whom we detected pivaloylcarnitine had a reduced concentration of free carnitine (C0) in blood which is explained by the massive renal loss of pivaloylcarnitine and not due to inhibition of free carnitine reabsorption by pivaloylcarnitine.^16^ Since there are reports that even short‐term administration of pivalic acid‐containing prodrugs can lead to symptoms (up to encephalopathy and hypoglycemia) from carnitine deficiency,^17^ this aspect should be further investigated in future‐targeted studies.

Our focus, however, was the dramatic impact of false‐positive NBS on families which has already been discussed in earlier publications.^18^, ^19^ A significant and persisting increase of parental anxiety as well as higher number of hospital admissions have been reported.^20^ Therefore, it is of utmost importance to apply second‐tier strategies to minimize false‐positive results; in the Hamburg lab this novel method has meanwhile been accredited and used without further actions or additional examinations in case of IVA exclusion and does not delay reporting of screening results.

The presented second‐tier UPLC‐MS/MS methods are very simple, highly selective, fast, and robust. In addition to detection of pivaloylcarnitine and differentiation from isovalerylcarnitine, they also allow identification of individuals with 2‐methylbutyryl‐CoA dehydrogenase deficiency (data not shown). The unlikely combination of the presence of either metabolic disorder and concomitant use of a pivalic acid‐containing drug should also be properly recognized. The most important hallmark of both methods is a simple dilution step after flow‐injection analysis and the approach is suitable for NBS labs using methods with or without derivatization. The costs of implementing the methods are marginal and limited to the purchase of an UPLC column and standard MS/MS solvents already available in MS/MS laboratories. The UPLC column used is extremely robust; the authors have been using their first column for more than 4 years, that is, for more than 2400 injections.

## 5 CONCLUSION

The methods described here can reliably identify false‐positive IVA results, consequently avoid further diagnostic steps, thereby additional costs and, most importantly, prevent unnecessary stress and anxiety of parents in a remarkably high number of cases. A further increase of false‐positive IVA cases has to be expected in the future. Therefore, either licensing of pivaloylester‐containing drugs for use during pregnancy in Germany should be reconsidered or a second‐tier method for C5 carnitine determination should be included in the NBS program.

## AUTHOR CONTRIBUTIONS

Simona Murko initiated the study, developed methods, wrote a first draft, and eventually finalized the article. Asra Dadkhah Aseman and Friederike Reinhardt prepared samples and performed the analyses. Gwendolyn Gramer helped interpreting data and editing the article. Jürgen G. Okun and Ulrike Mütze provided additional samples and data and were involved in editing the article. René Santer initiated the study, was involved in data interpretation and revised the article.

## FUNDING INFORMATION

Jürgen G. Okun and Ulrike Mütze are funded by the Dietmar Hopp Foundation, St. Leon‐Rot, Germany (2311 220 1DH1911376 to Georg F. Hoffmann).

## CONFLICT OF INTEREST

The authors declare that there is no conflict of interest that could be perceived as prejudicing the impartiality of the research reported.

## INFORMED CONSENT

All procedures were in accordance with the ethical standards of the responsible committee on human experimentation (institutional and national) and with the Helsinki Declaration of 1975, as revised in 2000. Informed consent was obtained from all patients for being included in the study.

## ANIMAL RIGHTS

This article does not contain any animal studies performed by any of the authors.

## Supporting information


**TABLE S1** Gradient used for NeoGram® derivatized kit.
**TABLE S2** Gradient used for NeoBase®2 nonderivatized kit.
**TABLE S3** Validation data for determination of C5 isomers using UPLC‐tandem mass spectrometry and NeoBase®2 kit.

## Data Availability

Data and material are available upon request from the corresponding author.
