# Supplementary material for: Neonatal screening for isovaleric aciduria: Reducing the increasingly high false‐positive rate in Germany
Source: JIMD Rep. 2022 Oct 28;64(1):114–20. doi: 10.1002/jmd2.12345 (PMC9830014; doi:10.1002/jmd2.12345)
Supplement: Supplementary file 1 — TABLE S1 Gradient used for NeoGram® derivatized kit. TABLE S2 Gradient used for NeoBase®2 nonderivatized kit. TABLE S3 Validation data for determination of C5 isomers using UPLC‐tandem mass spectrometry and NeoBase®2 kit. [file JMD2-64-114-s001.docx]

JIMD Reports=v.2022-09-16

**Supplementary Materials to**

**Neonatal screening for isovaleric aciduria:**

**reducing the increasingly high false-positive rate in Germany**

*Simona Murko ^1^, Asra Dadkhah Aseman ^1^, Friederike Reinhardt ^1^, Gwendolyn Gramer ^1^ , Jürgen G.Okun ^2^, Ulrike Mütze ^2^, René Santer ^1^*

^1^ Newborn Screening and Metabolic Laboratory, Department of Pediatrics, University Medical Center Eppendorf, Hamburg, Germany

^2^ Division of Child Neurology and Metabolic Medicine, Dietmar Hopp Metabolic Center, Center for Child and Adolescent Medicine Heidelberg, University Hospital, Heidelberg, Germany

**Corresponding Author:**

Dr. Simona Murko

Newborn Screening and Metabolic Laboratory

Dpmt of Pediatrics, Univ Med Center Eppendorf

Martinistraße 52, D - 20246 Hamburg,

Germany

Tel: +49-40-7410-57037

Fax: +49-40-7410-57318

Email: s.murko@uke.de

**Table S1:** Gradient used for NeoGram^®^ derivatized kit. Colums show percentage of solvent A (water with 0.1 % formic acid) and solvent B (acetonitrile with 0.1 % formic acid) at a given time. Injection volume 10 µl, flow rate 0.45 ml/min, run time 10 min.

*m/z* transitions: 302.2 🡪 85 for isomers and 311.2 🡪 85 for internal standard.

| Time | A (%) | B (%) |
| --- | --- | --- |
| Initial | 95 | 5 |
| 0.5 | 95 | 5 |
| 1.0 | 75 | 25 |
| 2.35 | 75 | 25 |
| 4.75 | 41 | 59 |
| 5.25 | 0 | 100 |
| 7.75 | 0 | 100 |
| 8.0 | 95 | 5 |
| 10.0 | 95 | 5 |

**Table S2.** Gradient used for NeoBase^®^2 non-derivatized kit. Colums show percentage of solvent A (water with 0.1 % formic acid) and solvent B (acetonitrile with 0.1 % formic acid): Injection volume 5 µl, flow rate 0.45 ml/min, run time 5 min.

*m/z* transitions: 246.1 🡪 85 for isomers and 255.2 🡪 85 for internal standard.

| Time | A (%) | B (%) |
| --- | --- | --- |
| Initial | 100 | 0 |
| 1.0 | 100 | 0 |
| 1.01 | 95 | 5 |
| 4.0 | 95 | 5 |
| 4.01 | 100 | 0 |
| 5.00 | 100 | 0 |

**Table S3.** Validation data for determination of C5 isomers using UPLC-MS/MS and Neobase^®^2 kit. Similar results were obtained using NeoGram^®^.

| **Isomer** | **R^2^** | **Level**  **(µmol/l)** | **Intra-assay**  **Variance**  **(%)** | **Inter-assay**  **Variance**  **(%)** | **Recovery**  **(%)** |
| --- | --- | --- | --- | --- | --- |
| Pivaloylcarnitine | 0.9992 | 1 | 7.8 | 9.7 | 110 |
|  |  | 10 | 4.4 | 10.4 | 105 |
| 2-Methylbutyrylcarnitine | 0.9987 | 1 | 5.9 | 11.5 | 100 |
|  |  | 10 | 7.9 | 9.3 | 108 |
| Isovalerylcarnitine | 0.9984 | 1 | 7.0 | 8.7 | 110 |
|  |  | 10 | 5.2 | 5.6 | 105 |
| Valerylcarnitine | 0.9993 | 1 | 1.2 | 12.1 | 100 |
|  |  | 10 | 8.6 | 9.1 | 109 |
